# “Carboranyl-cysteine”—Synthesis, Structure and Self-Assembly Behavior of a Novel α-Amino Acid

**DOI:** 10.1038/s41598-017-16926-w

**Published:** 2017-12-05

**Authors:** Tianyu He, Jennifer C. Misuraca, Rabi A. Musah

**Affiliations:** 1 Department of Chemistry, University at Albany, State University of New York, 1400 Washington Avenue, Albany, NY 12222 USA; 2 JEOL USA Inc, 11 Dearborn Rd, Peabody, MA 01960 USA

## Abstract

Substitution of the thiol proton in cysteine with *m*-carborane furnished 2-amino-3-(1,7-dicarba-*closo*-dodecacarboranyl-1-thio)propanoic acid (**3**), a boron cluster amino acid that exhibits self-assembly to form micron-sized constructs. Field emission scanning electron microscopy revealed that ethanol solutions of **3** form floret-shaped constructs, while fibrillar architectures are formed in water. Furthermore, slow evaporation of methanol solutions of **3** produced crystals whose structure was revealed by X-ray crystallography. The crystal structure shows that the hydrogen bonding interactions between pairs of **3** result in the formation of bilayers of 174 Å in length. The orientation of the clusters is not random in the crystal structure, such that the side-by-side aligned polyhedra are offset by 158 degrees. The material was characterized by FT-IR, NMR, high resolution mass spectrometry and dynamic light scattering. Circular dichroism studies indicated that self-assembly occurs at concentrations as low as 0.01 μM. This represents the first demonstration of self-assembly of a carborane-based molecule in the absence of metals. The amino acid motif provides opportunities for the controlled synthesis of extended multimeric units with tunable properties and the potential for applications in biology, medicine and materials chemistry.

## Introduction

Numerous biological processes are dependent upon the inherent ability of some molecules to spontaneously self-assemble into highly ordered constructs which confer functional attributes, or which serve as the etiological agents in disease progression and pathogenesis. Examples include α-hemolysin (Hla), a self-assembling extracellular protein that is an essential virulence factor for the pathogenesis of various *S*. *aureus* infections^1^, and amyloid-β peptides that self-assemble into fibrils and other forms, and which are responsible for the pathogenesis of Alzheimer’s disease^2^. A number of unnatural nucleic acid oligomers, peptides and proteins have also been observed to self-assemble into a variety of structures, thereby providing new approaches to the design of novel architectural frameworks with useful properties. For example, Ghadiri *et al*. demonstrated that short peptides can self-assemble into extended tubular β-sheet-like structures^3,4^, and Reches and Gazit showed that the simple di-peptide, Phe-Phe, could assemble into fibrils^5,6^. Subsequently, Ryan *et al*. reported that a fluorinated Fmoc protected phenylalanine (Fmoc-F5-Phe) self-assembled into hollow tubular constructs and formed a hydrogel at concentrations as low as 0.1 wt%^7^. With regard to free unprotected single amino acids, metal-promoted self-assembly has been known for quite some time^8–13^. However, it has been shown more recently that free aromatic amino acids in particular exhibit a proclivity for self-assembly that might have implications in biology and disease^2^. Gazit *et al*. demonstrated that aqueous solutions of free unprotected phenylalanine form amyloid-like fibrillary structures that may contribute to the etiology of phenylketonuria^14^. Tyrosine and tryptophan have also been shown to assemble into nano- and microscale architectures^15–17^.

These findings indicate that inherent in the amino acid scaffold are the fundamental attributes required for self-assembly. As such, this construct may provide unique opportunities for the introduction of non-proteinogenic moieties at the α-carbon that might yield novel self-assembling monomer units with unique and tunable properties. Non-proteinogenic α-amino acids are common in the plant kingdom where they often serve as chemical defense precursors that can be deployed on demand in response to tissue injury^18–20^. One such motif is that of the *S*-substituted cysteines and cysteine sulfoxides, which are degraded in response to tissue injury to form noxious volatiles with antimicrobial and herbivore deterrent properties. In these molecules, the thiol proton of cysteine is replaced by an alkyl or alkenyl group, although benzyl, pyrrolyl and pyridyl substituents have also been observed^21–24^. Thus, we considered cysteine to provide an entry point for the introduction of sulfur-bound unnatural moieties whose influence on the self-assembly properties of amino acids could be further studied. Here, we report on the results of the replacement of the thiol proton of cysteine with the boron cluster compound 1,7-dicarba-*closo*-dodecacarborane (C_2_H_12_B_10_, *m*-carborane). The resulting biomimetic construct spontaneously self-assembles into microscale superstructures.

## Results

### Compound Synthesis and Structural Characterization

**Figure 1:**
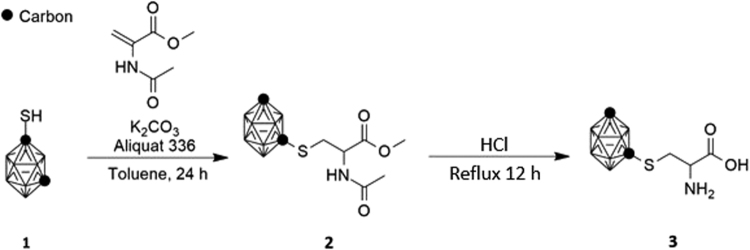
Synthetic route for 2-amino-3-(1,7-dicarba-*closo*-dodecacarboranyl-1-thio)propanoic acid (**3**). Michael addition of **1** to methy-2-acetamidoacrylate furnished **2**, which was hydrolyzed in aqueous acid to yield **3**.

The approach to the synthesis of 2-amino-3-(1,7-dicarba-*closo*-dodecacarboranyl-1-thio)propanoic acid (**3**) is shown in Fig. 1. Michael addition of *m*-carborane-1-thiol to methyl 2-acetamidoacrylate, followed by hydrolysis of the adduct under acidic conditions, furnished the *S*-substituted cysteine as a racemate. As anticipated, the IR spectrum showed a strong band at 2605 cm^−1^ representing B-H stretching, along with a characteristic doublet (3542 and 3457 cm^−1^), and peaks at 2680–3150 cm^−1^ and 1742 cm^−1^ for the –NH_2_, carboxyl and carbonyl moieties respectively. The high-resolution mass spectrum obtained from analysis by direct analysis in real time high-resolution mass spectrometry (DART-HRMS) is illustrated in the Supplementary Information Figure S1. The analysis was conducted under soft ionization conditions and thus, no fragmentation occurred. The spectrum features a distribution of peaks between nominal *m/z* 256 and 266 representing the protonated amino acid with varying distributions of ^11^B and ^10^B, with a [M + H]^+^ of 266.1989. An amino acid dimer exhibiting a distribution of peaks was also observed, centered at *m/z* 528.4039 (2[M + H]^+^).

The ^1^H and ^13^C NMR spectra acquired in methanol-*d*_4_ are shown in Supplementary Information Figure S2. The complexity of the ^1^H NMR spectrum of this relatively simple molecule is a consequence of the presence of both enantiotopic and diastereotopic protons (labeled a through e in the displayed structure). In the ^1^H NMR spectrum (Figure S2, Panel a), the multiplet representing the methine proton at the α-carbon appears at ~3.8 ppm (e) and the lone carborane C-H appears at 3.6890 ppm (a). The doublets that appear between 3.48–3.51 ppm are a consequence of the splitting of one of the methylene diastereotopic protons (c), by the α-carbon methine proton, while the signal for the second of the diastereotopic methylene protons (d) appears at 3.15–3.20 ppm. The unresolved signal from 1.8–2.3 ppm represents the protons (b) attached to boron in the carborane cluster, with the coupling of the boron and proton nuclei giving rise to the appearance of this signal^25,26^. The carboxylic acid and amino group protons are absent due to exchange with the solvent (methanol-*d*_4_). In the ^13^C NMR spectrum (Figure S2, Panel b), the carbonyl carbon appears at 169.7281 ppm, while the two carborane carbons appear at 71.2274 ppm (sulfur bound) and 56.5385 ppm. The aliphatic carbons of the CH and CH_2_ groups appear at 53.1038 ppm and 36.4231 ppm, respectively.

**Figure 2:**
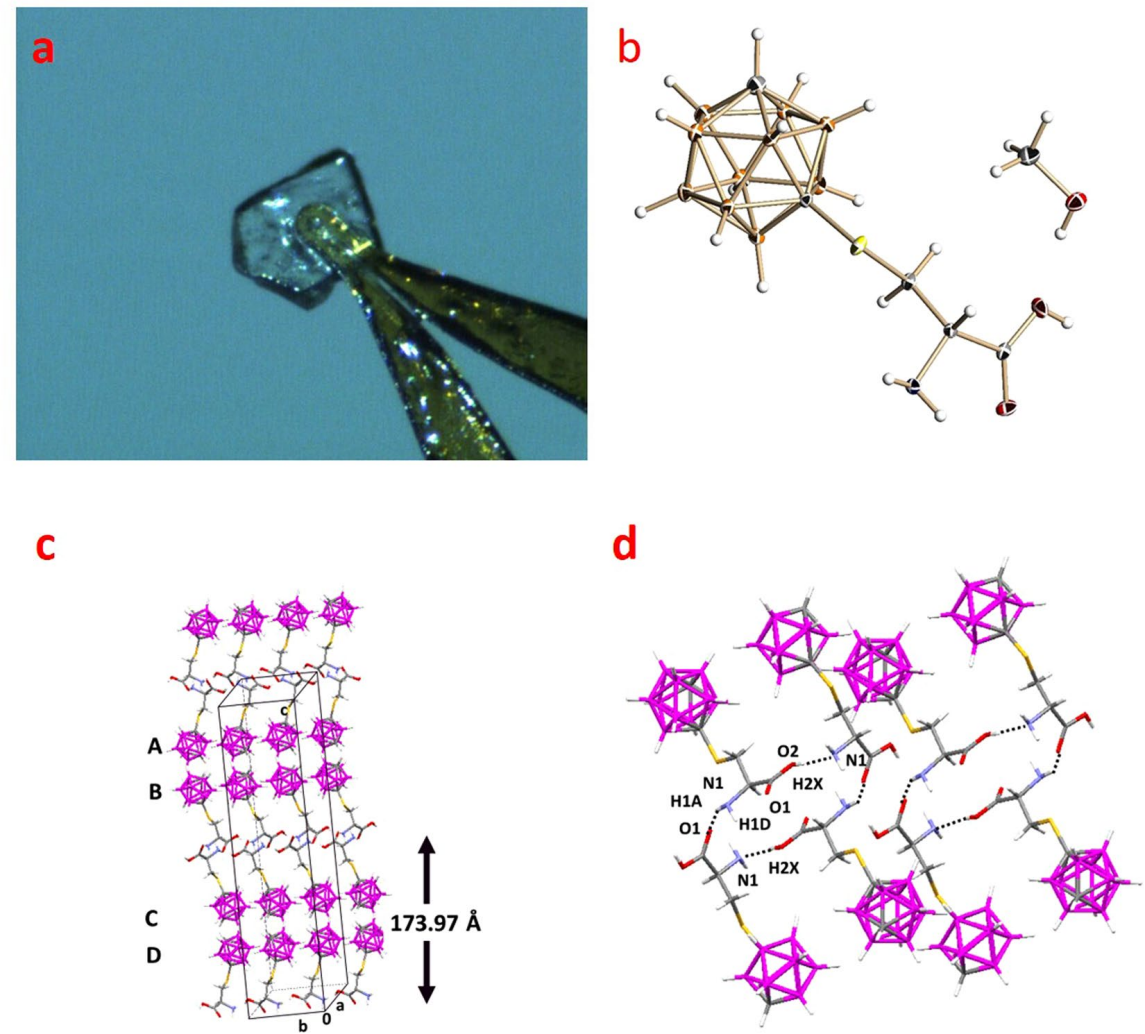
X-ray crystallography results for 2-amino-3-(1,7-dicarba-*closo*-dodecacarboranyl-1-thio)propanoic acid (**3**). Panel a, Crystal of **3** used for X-ray crystallographic analysis. Panel b, Solid state structure of **3**. Atoms are represented by thermal ellipsoids at the 40% probability level. Hydrogen atoms are represented by spheres of arbitrary radii. Only one orientation of the disordered groups is shown. Color code: orange, boron; grey, carbon; red, oxygen; white, hydrogen; yellow, sulfur. Panel c, Packing calculation for **3** revealed a composite of bi-layers of 173.97 nm in width with 4 different orientations designated as A, B, C and D. Panel d, Hydrogen bonding interactions of carboxyl side chains. Each molecule of **3** interacts with two adjacent molecules to establish a side-by-side arrangement for the bilayers.

X-ray quality colorless orthorhombic crystals of approximately 0.21 mm × 0.16 mm × 0.03 mm were obtained from saturated solutions of **3** in methanol-*d*_4_ at 25 °C under slow evaporation conditions over a period of ~5 weeks (Fig. 2, Panel a). High resolution diffraction studies were performed to determine the structure (Panel b) and packing arrangement of **3**. The packing of the molecules in the unit cell is shown in Fig. 2, Panel c. The molecules adopt a plate packing in four different layers (labeled A, B, C, and D), and are oriented as bilayers of 173.97 nm in width. The molecules were found to co-crystallize with the methanol-*d*_4_ solvent, whereby each methanol molecule was linked via hydrogen bonds with two carboranyl cysteines. Amino acid pairs were observed as hydrogen bonded dimers (Fig. 2, Panel d). The hydrogen bonds show D·····A distances significantly below the sum of the van der Waals radii (rw(N) + rw(O) = 3.07 Å) at 1.964 and 1.959 Å^27^, therefore representing moderately strong hydrogen bonds. Each unit cell has a volume of 142.2 Å and accommodates eight molecules of **3**. Among the intermolecular hydrogen bonding interactions observed were those between: (1) the O of the hydroxyl of **3** and the hydroxyl proton of methanol-*d*_4_; (2) the carboxylic acid proton of one molecule of **3** and the N of another molecule of **3**; and (3) the protons of the amino group with the methanol-*d*_4_ O. The average distance between the hydrogen bond donor and proton is 0.852 Å, while the average distance between the hydrogen acceptor and proton is 1.936 Å. Each bilayer is comprised of two rows of **3**.

Each carborane polyhedron is engaged in two types of interactions with adjacent carborane clusters: a side-by-side interaction, and a head-to-head interaction. Unlike the carbons in *m*-carborane which are indistinguishable because of the symmetry of the polyhedron, the bond to sulfur at C-1 made it possible to determine the orientation in space, relative to one another, of the carborane clusters. It was observed that despite the symmetry of the icosahedral cage and the free rotation that exists about the S-C bond, the orientation of the carborane clusters is not random in the crystal structure. Relative to one another, polyhedra that are aligned side-by-side are offset by 158.45 degrees, while at the same time, the C-H of each cluster is oriented towards the C-H of the adjacent one in each head-to-head arrangement involving the adjacent carboxylic acid. This pattern may reflect the fact that *o*- and *p*-icosahedral dicarba-*closo*-dodecaboranes exhibit a dipole the magnitude and direction of which is influenced by the orientation of the carbons relative to each other. The dipole moment of the *m*-carborane thiol has been estimated by both DFT and Hartree Fock methods using a 6–31 G basis set to be 1.06 D, with the positive end of the dipole moment concentrated between the two carbon atoms^28^. The location of the positive pole between the carbon atoms in each carborane cluster means that the positive end of one cluster is oriented towards the negative end of the other in the neighboring molecule. The presence of positive charge density at the relatively electronegative carbon atoms is attributed to charge transfer into vacant boron bonding orbitals^29^. The non-random orientation of the polyhedra relative to each other is suggestive of the presence of stabilizing dipolar interactions that may also influence directionality. Further computational studies are required to determine the relative magnitude of competing electronic factors such as polyhedron dipole versus hydrogen bonding interactions between individual amino acids and/or the solvent (and their influence on crystal packing orientation).

### FE-SEM and EDS Characterization of Self-assembly

**Figure 3:**
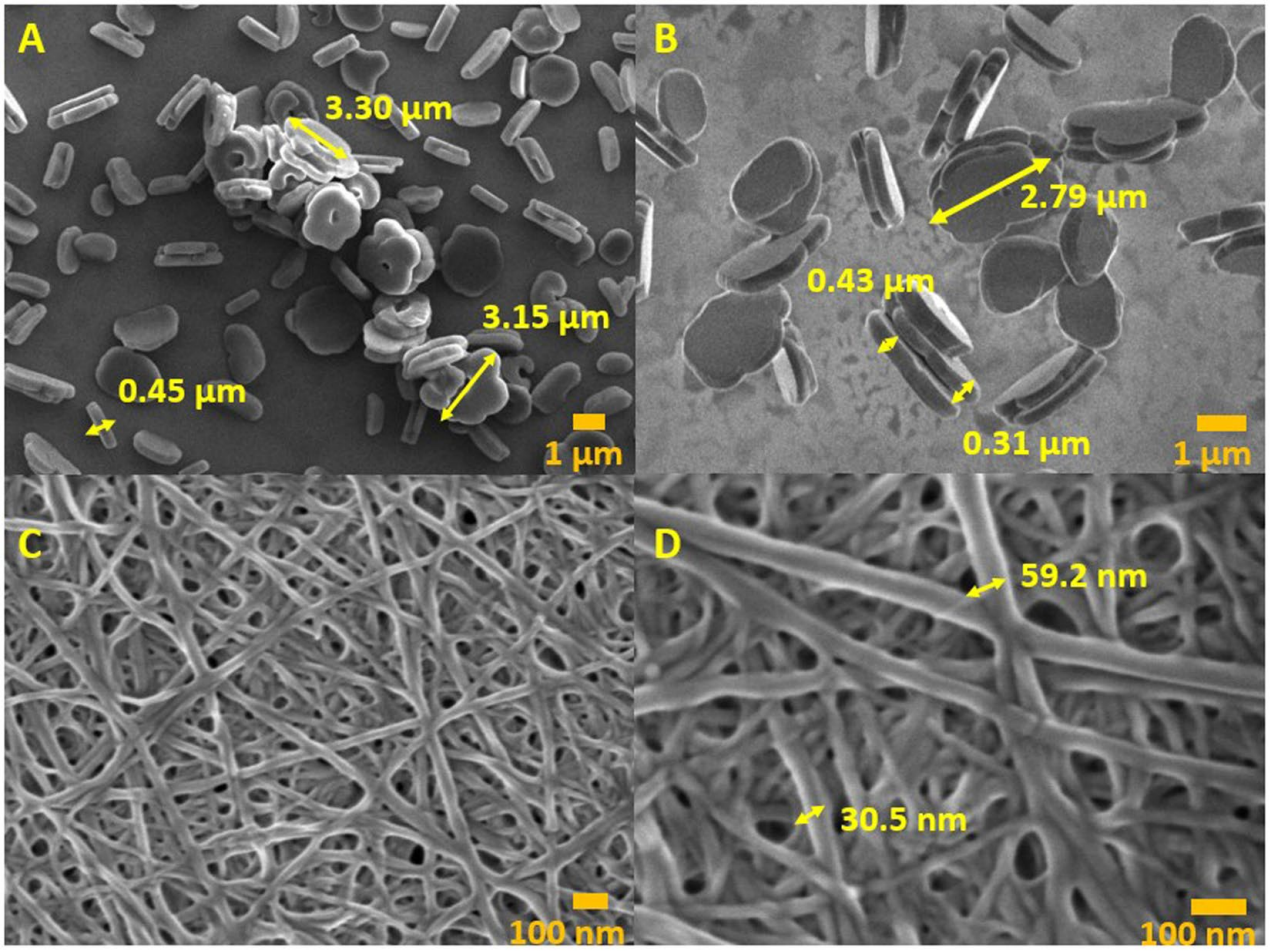
FE-SEM images of the self-assembled constructs formed from air evaporation of saturated ethanol (Panels a and b) and saturated water (Panels c and d) solutions of (**3**). Panels b and d each show magnifications of a segment of the images shown in Panels a and c, respectively. Panel a was imaged at 4 kV and Panels b, c, and d were imaged at 0.8 kV.

**Figure 4:**
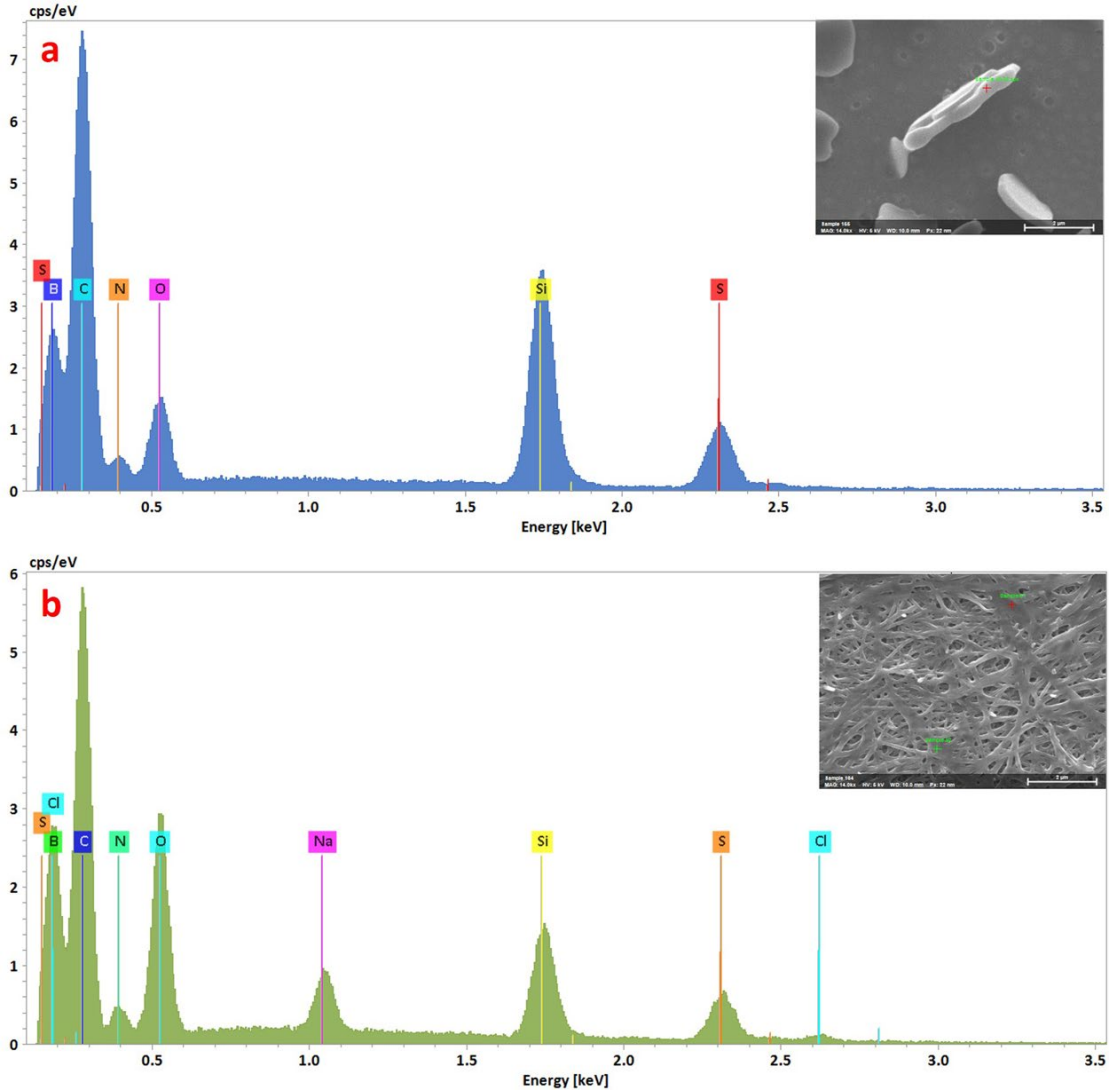
Electron dispersive spectra of the self-assembled “florets” and fibrils shown in Fig. 3 Panels a and c, respectively. The spectra showed that the self-assembled constructs contained B, C, N, O, and S.

**Figure 5:**
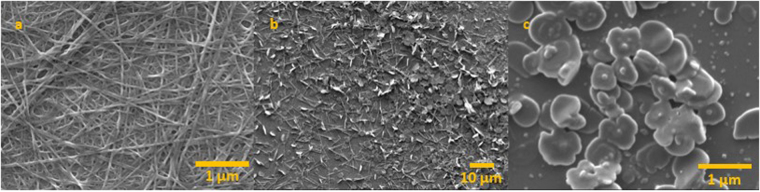
FE-SEM images of the self-assembled constructs formed from air evaporation of a water solution (0.25 mg/mL) (Panel a). After resuspension in ethanol, a transition from fibrils to florets was revealed (Panel b). Panel c shows a magnified section of the image shown in Panel b.

Despite the reliable formation of X-ray quality crystals from solutions of **3** during slow evaporation of methanol-*d*_4_, attempts at crystallization from water were unsuccessful. Instead, aqueous and ethanol solutions of **3** furnished micron-sized constructs. Thus, imaging by FE-SEM revealed that air evaporation of ethanol from a 0.10 mg/mL solution of **3** under ambient conditions resulted in circular floret-shaped disks (Fig. 3, Panels a and b) with smooth surfaces. Many are characterized by having 5–6 sides or “petals” with an indentation or hole at the center. They range in length from between ~3–3.5 µm, with a depth of ~0.3–0.4 µm. Where an inner hole appears, its diameter is ~0.7 µm. They are attracted to one another, as implied by their tendency to stack. On the other hand, samples prepared by air evaporation of water from a 0.25 mg/mL solution of **3** resulted in the formation of fibrils. These ranged from a width of 30–50 nm and were up to several microns in length (Fig. 3, Panels c and d). The fibril constructs appear similar to those associated with hydrogels formed from Fmoc protected phenylalanine (Fmoc-F5-Phe), and **3** was also observed to form a hydrogel under slow evaporation of water from aqueous solutions^7,10,30^. For both the fibrils and the florets, EDS analysis confirmed the presence of the constituent elements, including sulfur, boron, nitrogen and oxygen (Fig. 4). Furthermore, resuspension into ethanol of fibrils formed from evaporation of water, followed by subsequent evaporation of the ethanol, revealed a transition from fibrils to florets (Fig. 5). This indicates that the formation of the fibrils is not a result of covalent modification of the amino acid, and can be reversed when the solvent is changed. Self-assembled constructs were not observed when solvent was evaporated from solutions of **3** in *n*-propyl- or *n*-butyl alcohols. Compound **3** was insoluble in non-polar solvents (benzene, hexane, toluene).

### Attempted TEM characterization of self-assembled architectures

An attempt was made to characterize the self-assembled constructs by TEM. The ethanol and water solutions of compound **3** were deposited on copper grids with mesh and polymer supports. The grids were allowed to air dry for 24 h before imaging. Using this substrate, TEM imaging revealed micron-sized self-assembled constructs. Selected TEM images are provide in Supplementary Information Figure S3. Evaporation of water from aqueous solutions of compound **3** resulted a porous film, while evaporation of ethanol from solutions of **3** revealed stacked architectures of the “florets”.

### Solubility and FT-IR studies of compound 3

The solubility of compound **3** in methanol, ethanol and water at room temperature was determined to gain insights into the relationship between solubility and the morphologies observed in different solvents. The solubility of compound **3** in water, ethanol, and methanol was determined to be 0.25 mg/mL, 10 mg/mL and 4.9 mg/mL, respectively. In order to gain insight into the differences in inter- or intramolecular interactions that resulted in the solvent-dependent differences in architectures, FT-IR measurements on the materials formed in different solvents were performed. The results are shown in Supplementary Information Figure S4. The three spectra are essentially superimposable except in the carbonyl region, where carbonyl absorptions of 1618 cm^−1^, 1611 cm^−1^, and 1607 cm^−1^ were observed for water, ethanol and methanol respectively.

### Dynamic light scattering (DLS) analysis

**Figure 6:**
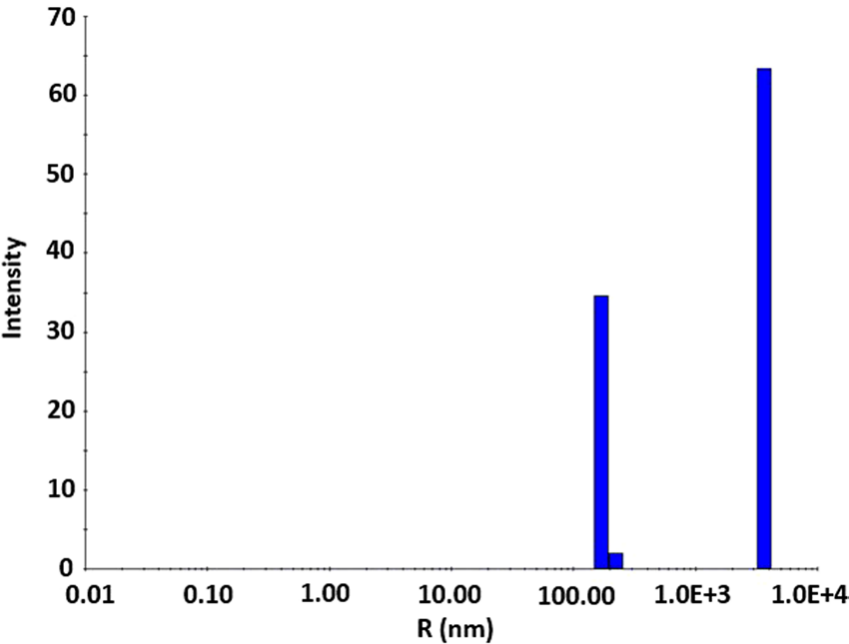
Dynamic light scattering (DLS) analysis of an ethanol solution of **3**. The majority (99.98250%) of the particles have a universal size of 173.53 nm (radius). The polydisperse distribution (PD) of 7.2% indicates that the particles have a single radius value (PD values of no more than 15% indicate single sized particles).

DLS studies were performed to determine the size distribution profile of the constructs of **3** that were formed from evaporation of the solvent from ethanol and water solutions. The results for the ethanol solution are shown in Fig. 6. The majority (99.98%) of the particles in the solution have a universal size of 173.53 nm (radius) with a polydisperse distribution (PD) of 7.2%. However, the fibrillar (as opposed to particulate) nature of the particles formed in water precluded extraction of results by this method.

### Circular dichroism (CD) studies

**Figure 7:**
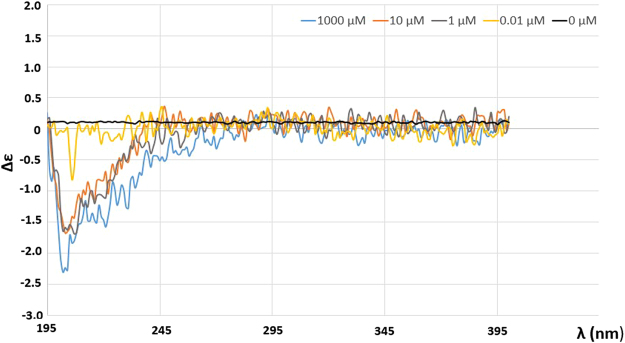
Circular dichroism spectra of water solutions of 2-amino-3-(1,7-dicarba-*closo*-dodecacarboranyl-1-thio)propanoic acid at concentrations ranging from 0–1000 µM. Evidence of the formation of secondary structure occurred at a threshold concentration of 0.01 μM. Spectra of 1, 10 and 1000 μM solutions showed dramatic negative Δε centered at 202 nm.

CD analysis was performed on **3** that was solubilized in both ethanol and water, in order to determine the approximate concentration at which the self-assembly occurs. Like other α-amino acids containing a single chiral center, Cotton effects (CE) between 200–300 nm for the *S* and *R* enantiomers would be anticipated, with their CD spectra being mirror images of one another. Thus, racemates such as **3** used in this study would be expected to exhibit no chiroptical signal until self-assembly ensues. CD spectra of increasing concentrations of **3** in water (0–1000 μM, Fig. 7) yielded results consistent with this prediction. Thus, while a 0.01 μM solution of **3** exhibited a flat line indicative of a racemate, spectra of 1, 10 and 1000 μM solutions showed a dramatic negative change in optical rotatory dispersion (Δε = −1.51, −1,65, −2.30, respectively) centered at 202 nm, despite the fact that **3** is a racemate. This result indicates that at a concentration of between 0.1 and 1 μM, solutions of **3** in water begin to adopt ordered structure, even though the individual amino acids are not covalently linked. Similar experiments conducted on **3** in ethanol (Supplementary Information Figure S5) revealed that the wavelength of maximum Δε is slightly red-shifted relative to that in water (209 nm vs 202 nm respectively). HT curves and UV spectra were measured for all solutions (see Supplementary Information Figures S6-S9) to confirm the validity of the CD results.

## Discussion

Hohman *et al*. showed recently that carboranethiol isomers self-assemble on gold, with the orientation of the molecules on the gold surface being controlled by the direction of the dipole of the carborane thiol relative to the gold surface^28^. However, the work described here represents the first report of the spontaneous self-assembly of a carborane-containing compound in the absence of metals. The integration of the carborane into cysteine to create a molecule analogous to the plant chemical defense precursors found in some *Allium*^21^ and *Phytolacacceae*^20^ genus plants, results in a carboranyl-L-cysteine which assembles into microscale-sized architectures the nature of which are solvent dependent. Rapid evaporation of ethanol solutions furnishes florets, while slow evaporation from the same solvent yielded crystals (data now shown). Aqueous solutions of compound **3** resulted in the formation of fibrils. The X-ray crystal structure indicated the presence of hydrogen bonding interactions between the carboxyl groups of amino acid pairs, which resulted in the formation of bilayers of alternating polyhedra oriented in a head-to-head arrangement.

In contrast to simple boron hydrides such as BH_3_ and B_2_H_6_ which are highly reactive as well as air- and moisture-sensitive, the neutral hydrophobic *closo*-dodecacarboranes exhibit high kinetic and thermal stability. This has been attributed to the three-dimensional delocalization (pseudo-aromaticity) of the σ-framework electrons^31,32^. The volume of the polyhedral cage (70–80 Å) is similar to the spherical volume created by the rotational sweep of the phenyl ring^33,34^. Therefore, it is perhaps not surprising that the crystal packing revealed by the X-ray data, is analogous to that observed in phenylalanine^35^. In the case of phenylalanine, the packing is stabilized by alternating hydrophobic and hydrophilic interactions to form a layered structure. Two rows of phenylalanine molecules are held together by hydrogen bonding and phenyl ring stacking interactions. The combination of hydrogen bonding and stacking interactions are a key contribution to the self-assembly of phenylalanine. Similarly, in compound **3**, each layer is composed of two rows of carboranyl cysteine molecules connected via hydrogen bonds, while the inter-layer junctions occur through edge-to-edge interactions between the hydrophobic carborane clusters (pseudo aromatic environment). Relative to one another, the carborane clusters are arranged such that the electron deficient face of one is oriented towards the electron-rich face of the adjacent one. The extreme hydrophobicity of the clusters coupled with the highly polar solvent conditions may promote the observed self-assembly via a type of stacking interaction involving the carborane clusters.

The results of SEM and EDS experiments demonstrated that there is no difference in the molecular composition of the constructs when the solvent is changed, and the reversible transition of the constructs from fibrils to florets indicates that the formation of the constructs is not a consequence of covalent modification.

The SEM experiments revealed the dimensions of the “floret” constructs to be 0.3–0.4 μm in depth with a length of 3–3.5 μm and the diameter of the inner hole of 0.7 μm. This was in contrast to the dimensions determined by DLS, which revealed uniformly sized particles with a diameter of ~173 nm, which is several orders of magnitude smaller. We attribute this disparity to be a consequence of the differences, necessitated by the requirements of the experimental techniques used, in how the samples were derived. While the DLS experiments were performed on solutions with a maximum concentration of 10 mg/mL (the highest concentration that could be achieved without precipitation), the SEM experiments were conducted under high vacuum on samples that were acquired by evaporation of the solvent from prepared solutions. The increases in concentration that occurred with solvent vaporization result in increased aggregation with commensurate increases in particle size. The particles observed by DLS may serve as nucleation sites for the agglomeration that results in the formation of the discs observed by SEM. This hypothesis is the subject of further investigations in our laboratory.

The FT-IR spectra of compound **3** from water, ethanol and methanol showed that the carbonyl absorptions were affected, while the remaining areas of the spectra were essentially superimposable, even in the fingerprint region. FT-IR carbonyl stretching frequencies have been noted to serve as reporters of hydrogen-bonding interactions, with increasing hydrogen bonding being reflected by decreasing absorptions^36^. The carbonyl stretching frequencies observed here (i.e. 1617 cm^−1^, 1611 cm^−1^, and 1607 cm^−1^ for water, ethanol and methanol respectively), coupled with the difference in the architectures observed in water, ethanol and methanol (fibrils, florets and a fine powder respectively) imply that the nature of the constructs formed is associated with differences in hydrogen bonding interactions at the carbonyl in each case.

Circular dichroism studies showed that self-assembly of **3** in solution occurs at concentrations between 0.1 and 1 μM, and that the size of the constructs formed was not a function of concentration. Despite the compound being a racemate, the Cotton effect observed for both water and ethanol solutions of compound **3** was very informative, and yielded results that could not have been acquired had individual enantiomers been analyzed. The results showed that below a concentration of 0.1 μM there was no evidence of self-assembly and the absence of optical rotatory dispersion (flat line) is exactly what would be anticipated for a racemic mixture. However, at a concentration of > 0.1 μM, a negative Cotton effect was observed, marking the formation of ordered structures (self-assembly). Furthermore, at the minimum concentration at which self-assembly occurred, the observed CD spectra remained similar for higher concentrations, indicating that there was no change in structure as a function of concentration. The results of dynamic light scattering experiments revealed that > 99% of the particles have a uniform size. Several reports have appeared illustrating the integration of *closo*-carboranes into biological constructs^37–57^, but it remains unclear whether these molecules exhibit self-assembly properties. However, as shown here, integration of a carborane into an α-amino acid motif results in a molecule that displays self-assembly, and provides opportunities for the synthesis of extended multimeric units with tunable properties and potential for application in biology, medicine and materials chemistry.

## Methods

### Materials

Methyl 2-acetamidoacrylate, *m*-carborane-1-thiol, benzene-*d*_6_, methanol-*d*_4_ anhydrous diethyl ether, *n*-butyl alcohol, and Aliquat 336 were purchased from Sigma Aldrich (St. Louis, MO). Toluene, *n*-propyl alcohol and Na_2_SO_4_ were purchased from Fisher Scientific (Waltham, MA) and K_2_CO_3_ was purchased from VWR (Radnor, PA). Ethanol (200 proof) and isopropyl alcohol were purchased from Pharmco Aaper (Brookfield, CT). All the solvents were used as received without further purification.

### FT-IR spectroscopy

IR spectra were acquired using a PerkinElmer FT-IR Spectrum Two spectrometer (Waltham, MA). PerkinElmer Spectrum 10 software was used for data processing.

### NMR spectroscopy

^1^H and ^13^C NMR spectra were acquired using a Bruker 500 MHz AVIIIHD spectrometer (Billerica, MA, USA) with a PRODIGY BBO probe installed. Topspin 3.5 pl.6 software was used to process the data. Chemical shifts are reported in δ using residual solvent as the internal standard.

### Direct Analysis in Real Time-High Resolution Mass Spectrometry (DART-HRMS)

All mass spectra were acquired in positive ion mode using a DART-SVP^TM^ ion source (Ionsense, Saugus, MA, USA) coupled to a JEOL AccuTOF high resolution mass spectrometer (JEOL USA, Inc., Peabody, MA, USA). The ion source was operated with ultra-high purity helium (Airgas, Albany, NY, USA) at a flow rate of 2 L/min and a temperature of 350 °C. Poly(ethyleneglycol) (PEG; average MW 600) was analyzed with every acquired spectrum as a standard for accurate mass determinations. TSSPro 3 software (Shrader Analytical, Detroit, MI, USA) was used for data processing including averaging, centroiding and background subtraction.

### Melting point determination

A MEL-TEMP capillary melting point apparatus (Sigma Aldrich, St. Louis, MO) was used to determine the melting points.

### Synthesis of 2-acetamido-3-(1,7-dicarba-*closo*-dodecacarboranyl-1-thio)propanoic acid methyl ester (2)

Compound **2** was synthesized by Michael addition of 1,7-dicarba-*closo*-dodecacarborane-1-thiol to methyl 2-acetamidoacrylate. This was followed by acid-catalyzed hydrolysis. Briefly, 1,7-dicarbadodecaborane-1-thiol (500 mg, 2.83 mmol), methyl 2-acetamidoacrylate (289 mg, 2.02 mmol), Aliquat 336 (64 mg 0.16 mmol), and K_2_CO_3_ (84 mg, 0.61 mmol) were suspended in 40 mL of toluene in an Erlenmeyer flask. After stirring at room temperature for 24 h, the mixture was washed with water and the organic layer was dried over anhydrous Na_2_SO_4_, filtered and evaporated to yield a white solid. The crude product was purified by preparative TLC using 20 × 20 cm silica UNIPLATE-T Taper Plates (Analtech, Newark, DE), with anhydrous diethyl ether as the mobile phase. Compound **2** appeared as a white solid (396 mg, 1.24 mmol, yield 61%, m.p. 91–93 °C). DART-HRMS: *m/z* = 322.2259 ([M + H]^+^). FTIR: υ (cm^−1^) 3278 (m, N-H), 3061 (m, C_cluster_-H), 2998 and 2954 (m, C_alkyl_-H), 2600 (s, B-H), 1744 and 1667 (s, C = O). ^1^H NMR (C_6_D_6_), δ_H_ 6.07 (d, 1 H, *J* = 7.2 Hz), 4.80 (q, 1 H, *J* = 6 Hz), 3.243 (s, 3 H), 2.98 (q, 2 H, *J* = 6 Hz), 1.54 (s, 3 H), 1.78–3.33 (m, cluster H). ^13^C NMR (C_6_D_6_), δ_C_ 170.4, 169.5, 72.1, 55.9, 52.2, 51.7, 38.37, 22.36. ^11^B NMR (C_6_D_6_), δ_B_ −2.57 and −3.80 (1B), −9.42 and −10.57 (5B), −12.36, −13.68, −15.08 (4B)

### Synthesis of 2-amino-3-(1,7-dicarba-*closo*-dodecacarboranyl-1-thio)propanoic acid (3)

Compound **2** (396 mg, 1.24 mmol) was added to 52 mL of 0.6 M HCl and the solution was refluxed for 12 h. The reaction mixture was filtered and the filtrate was lyophilized. The residual dried white solid was then recrystallized from hot water to yield 2-amino-3-(dodecaboranyl-1-thio)propanoic acid (194.5 mg, 0.74 mmol, yield 60%, m.p. 173 °C). DART-HRMS: *m/z* = 266.1989 ([M + H]^+^). FTIR: υ (cm^−1^) 3542 and 3457 (w, NH_2_), 3105–2760 (b, O-H), 2605 (s, B-H), 1742 (s, C=O). ^1^H NMR (CD_3_OD), δ_H_ 3.82 (q, 1 H, *J* = 3.8 Hz), 3.69 (s, 1 H), 3.49 (dd, 1 H, *J* = 3.3, 10.8), 3.243 (dd, 1 H, *J* = 3.7, 10.8), 1.75–2.30 (m, cluster H). ^13^C NMR (CD_3_OD), δ_C_ 169.7, 71.2, 56.5, 53.1, 36.4. ^11^B NMR (CD_3_OD), δ_B_, −3.2 and −4.22 (1B), −10.10 and-11.00 (5B), −12.59, −13.68, −14.93 (4B)

### Crystal structure determination

Crystallization was accomplished by slow evaporation in methanol-*d*_4_ over a period of 5 weeks at 4 °C. A 2 mL methanol-*d*_4_ solution (1.0 mM) of **3** was prepared, filtered, and then transferred to a 20 mL scintillation vial which was loosely capped. The vial was allowed to stand at 4 °C undisturbed until crystals were observed. Data collection was performed on a Bruker D8 VENTURE X-ray diffractometer with a PHOTON 100 CMOS detector equipped with a Mo-target X-ray tube (λ = 0.71073 Å) at T = 100(2) K. The single crystal was mounted on a MiTeGen crystal holder (20 mm). A crystal-to-detector distance of 60 mm and exposure time of 20 s per frame (0.5° for each frame) were used for data collection. Data reduction and integration were performed with the Bruker software package SAINT (version 8.37 A). Data were corrected for absorption effects using the empirical method as implemented in SADABS (version 2016/2). The structure was solved by SHELXT9 and refined by full-matrix least-squares procedures using the Bruker SHELXTL (version 2016/6) software package^58,59^. All non-hydrogen atoms were refined anisotropically. Hydrogen atoms attached to N1, O2 and O3 were found in the difference Fourier map and refined independently. All other H-atoms were also included at calculated positions and refined as riders, with U_iso(H)_ = 1.2 U_eq_ and U_iso(H)_ = 1.5 U_eq_ for methyl groups. A detailed presentation of the crystallographic data is presented in the Supporting Information section. The crystal data, data collection and structure refinement information are outlined in Supplementary Information Table S1. The selected bond lengths and angles and hydrogen bonding interactions are outlined in Supplementary Information Tables S2 and S3, respectively. The crystal structure is shown in Fig. 2 (Panel a).

### Field emission scanning electron microscopy (FE-SEM) and energy dispersive X-ray spectroscopy (EDS)

FE-SEM images were acquired using a JEOL JSM-7200F scanning electron microscope (JEOL USA, Inc, Peabody, MA). Silicon wafers (4” silicon 5 × 7 chips), carbon conductive tape (double coated, 8 mm W × 20 mm L) and SEM stubs were purchased from TED Pella, Inc (Redding, CA). The silicon wafers were affixed to the SEM stubs using carbon conductive tape. Saturated solutions of **3** in various solvents including water, methanol, ethanol, *n*-propyl alcohol and *n*-butyl alcohol (5 μL) were deposited on 4” silicon 5 × 7 chips and the samples were either air dried, or the solvent was evaporated under house vacuum within a desiccator. The wafers were imaged by FE-SEM and then coated with platinum using a Quorum Q150T ES turbo-pumped sputter coater (Guelph, ON, Canada) before analyzing with a 60 mm^2^ XFlash Bruker EDS detector.

### Transmission electron microscopy (TEM)

TEM images were acquired using a JEOL 2010F field emission TEM operated at 200 kV. Bright imaging and electron diffraction modes were used to conduct the imaging experiments. Saturated solutions of **3** in ethanol and water (1 µL) were deposited onto 3 mm copper grids with mesh and polymer support (SPI supplies, West chester, PA). The grids were allowed to dry under ambient condition for 24 h before imaging.

### Solubility and IR studies

Solubility was determined by adding solvents in 100 µL increments until the compound was fully dissolved at room temperature. A Fisher Scientific FS30 Ultrasonic Cleaner (Hampton, NH) was used to sonicate the mixtures. The methanol, ethanol and water solutions of compound **3** were then dried using the same protocol used in the SEM studies. The dried material was analyzed by FT-IR using a PerkinElmer FT-IR Spectrum Two spectrometer (Waltham, MA). PerkinElmer Spectrum 10 software was used for data processing.

### Circular dichroism (CD) spectroscopy

All CD spectra were recorded using a JASCO J-815 CD spectrometer (Oklahoma City, OK). CD spectra were collected over a range of compound concentrations (0 μM-1000 μM in water and ethanol). Spectra were collected over a scan range of 195–400 nm at 25 °C in a 0.01 cm path-length cuvette. The band width was 1.00 nm with a scanning speed of 100 nm/min. Data processing, including background subtraction and data smoothing, were performed using Spectra Manager Version 2 software (JASCO, Oklahoma City, OK).

### Dynamic light scattering (DLS)

DLS measurements were conducted using a DynaPro™ Dynamic Light Scattering (DLS) Instrument with temperature control (Wyatt Technology, Goleta, CA). A 5 mL ethanol solution of **3** was prepared, filtered, and 12 µL of the solution was transferred into a 12 μL 8.55 mm center height quartz cuvette chamber (Wyatt Technology, Goleta, CA) using standard pipettors with gel loading tips. The cuvette was covered with a plastic cap to prevent evaporation, and the absence of bubbles in the sample chamber was confirmed visually through the cuvette window. Manufacturer-supplied Dynamics software (version 6.7.7.9) was used for operating the instrument as well as for data processing. The same protocol was used to conduct experiments with water solutions of compound **3**.

### Data availability

All data generated or analyzed during this study are included in this published article (and its Supplementary Information files).

## Electronic supplementary material

Supplementary Information
