# Author Correction: “Carboranyl-cysteine”—Synthesis, Structure and Self-Assembly Behavior of a Novel α-Amino Acid

**DOI:** 10.1038/s41598-018-23184-x

**Published:** 2018-03-19

**Authors:** Tianyu He, Jennifer C. Misuraca, Rabi A. Musah

**Affiliations:** 1 Department of Chemistry, University at Albany, State University of New York, 1400 Washington Avenue, Albany, NY 12222 USA; 2 JEOL USA Inc, 11 Dearborn Rd, Peabody, MA 01960 USA

Correction to: *Scientific Reports* 10.1038/s41598-017-16926-w, published online 05 December 2017

The Acknowledgements section in this Article is incomplete.

“The financial support of the National Science Foundation to R.A.M. (grants 1310350 and 1429329), is gratefully acknowledged. Thanks are extended to Dr. Zheng Wei for assistance with X-ray crystal structure determination and Prof. Kathy Dunn at SUNY Polytechnic Institute for assistance with TEM imaging.”

should read:

“The financial support of the National Science Foundation to R.A.M. (grants 1310350, 1429329 and 1710221), is gratefully acknowledged. Thanks are extended to Dr. Zheng Wei for assistance with X-ray crystal structure determination and Prof. Kathy Dunn at SUNY Polytechnic Institute for assistance with TEM imaging.”

