# Supplementary material for: “Carboranyl-cysteine”—Synthesis, Structure and Self-Assembly Behavior of a Novel α-Amino Acid
Source: Sci Rep. 2017 Dec 5;7:16995. doi: 10.1038/s41598-017-16926-w (PMC5717241; doi:10.1038/s41598-017-16926-w)
Supplement: Supplementary file 1 — Supplementary Information [file 41598_2017_16926_MOESM1_ESM.pdf]

“Carboranyl-cysteine”—Synthesis, Structure, and Self-Assembly Behavior of a Novel α-Amino Acid

Tianyu He[1], Jennifer C. Misuraca[2] and Rabi A. Musah[1]*

[1]Department of Chemistry, University at Albany, State University of New York, 1400 Washington Avenue, Albany, NY 12222, USA

[2]JEOL USA Inc, 11 Dearborn Rd, Peabody, MA 01960, USA

*Correspondence should be addressed to R.A.M (rmusah@albany.edu)

SUPPPPLEMENTARY INFORMATION

This document contains: (1) the high-resolution mass spectrum of 2-acetamido-3-(1,7-dicarba-*closo*-dodecacarboranyl-1-thio)propanoic acid; (2) TEM micrographs of compound **3**; (3) IR spectra of compound **3**; (4) circular dichroism spectra of compound **3** in ethanol at concentrations ranging from 0 μM to 1000 μM; (5) the HT curve and absorbance curve of all the CD spectra collected; (6) a description of the general procedure for crystal growth; and (7) tables containing information on the experimental details of the X-ray crystal structural determination experiment, selected bond lengths and angles, and hydrogen bonding interactions.

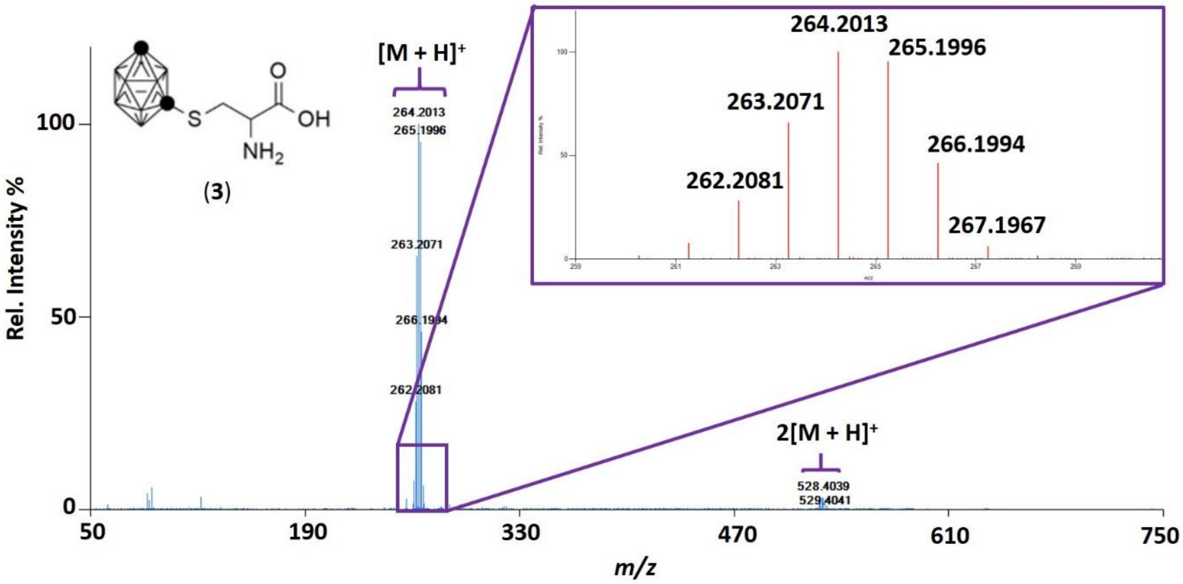

Figure S1. Direct analysis in real time-high resolution mass spectrum (DART-HRMS) of (**3**). This polyisotopic mass spectrum exhibits the statistical distribution of ^{11}B and ^{10}B which occurs naturally in a ratio of $^{11}B/^{10}B = 4.0$. This distribution shows the range of peaks from nominal *m/z* 256-267, with each peak in the cluster representing the protonated form of the corresponding monomer unit.

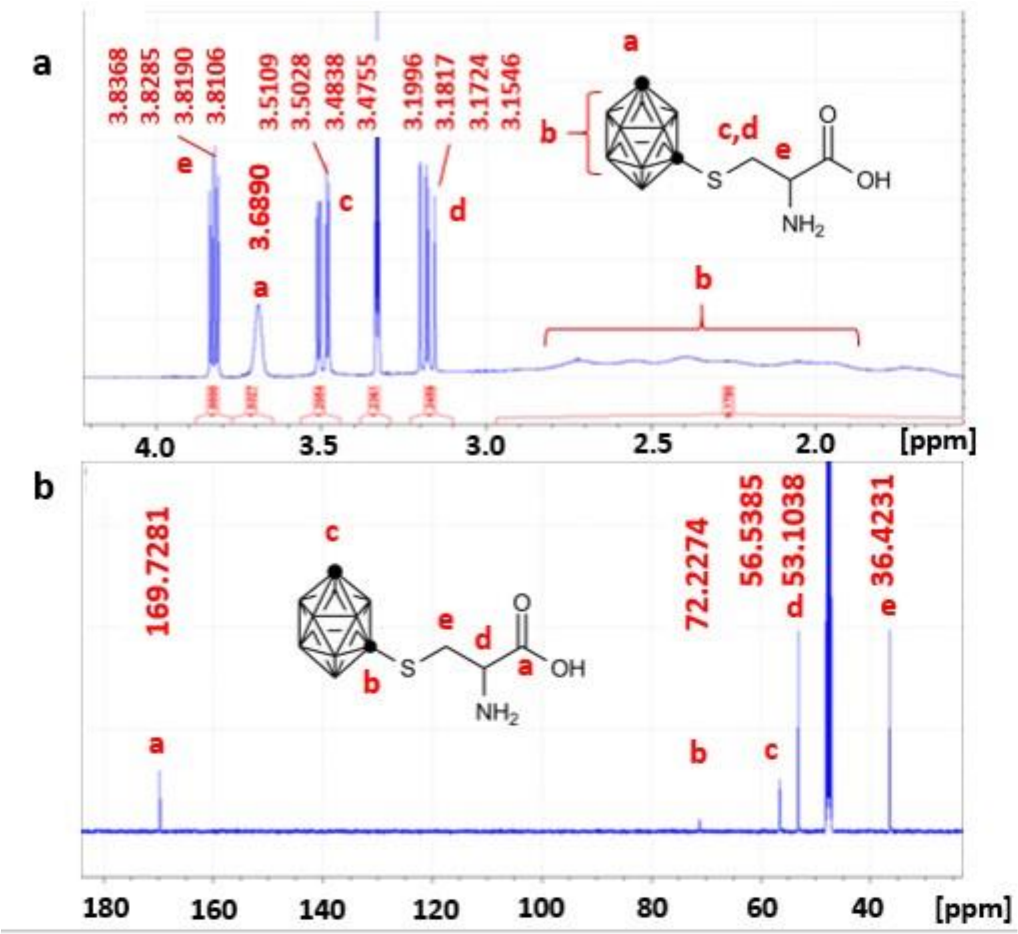

Figure S2. Panel a: ^{1}H NMR spectrum of 2-acetamido-3-(1,7-dicarba-*closo*-dodecacarboranyl-1-thio)propanoic acid (**3**). Panel b: ^{13}C NMR spectrum of 2-acetamido-3-(1,7-dicarba-*closo*-dodecacarboranyl-1-thio)propanoic acid (**3**). The peak assignments are indicated.

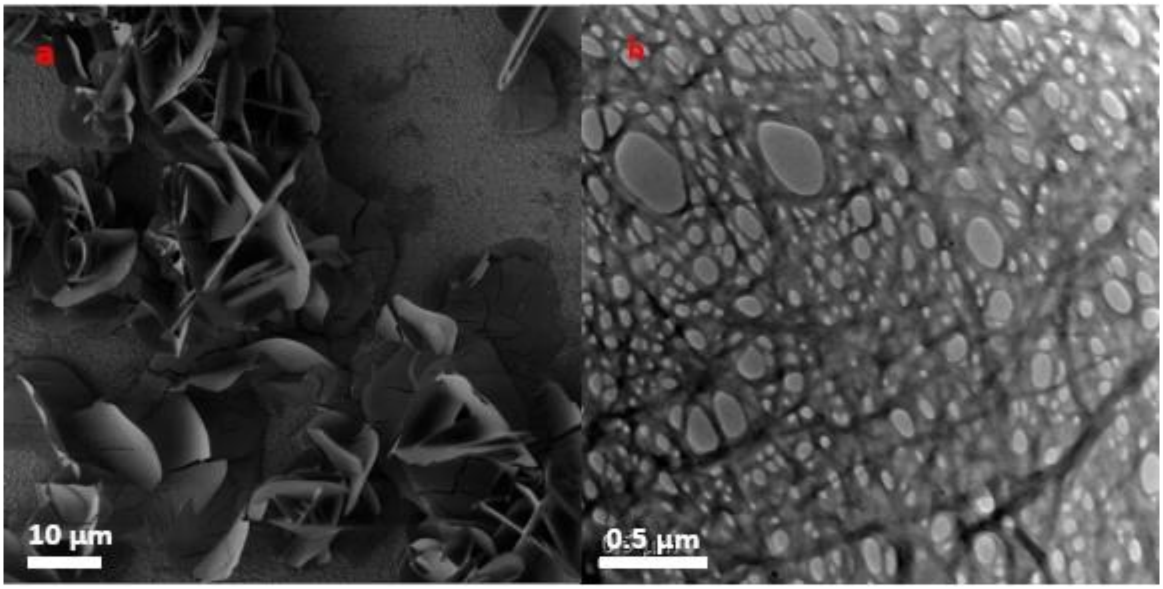

Figure S3. TEM images of compound **3** in saturated ethanol (Panel a) and saturated water (Panel b) solutions. Stacking of the floret constructs was observed when ethanol was evaporated from solutions of **3**. A porous film was observed when water was evaporated from aqueous solutions.

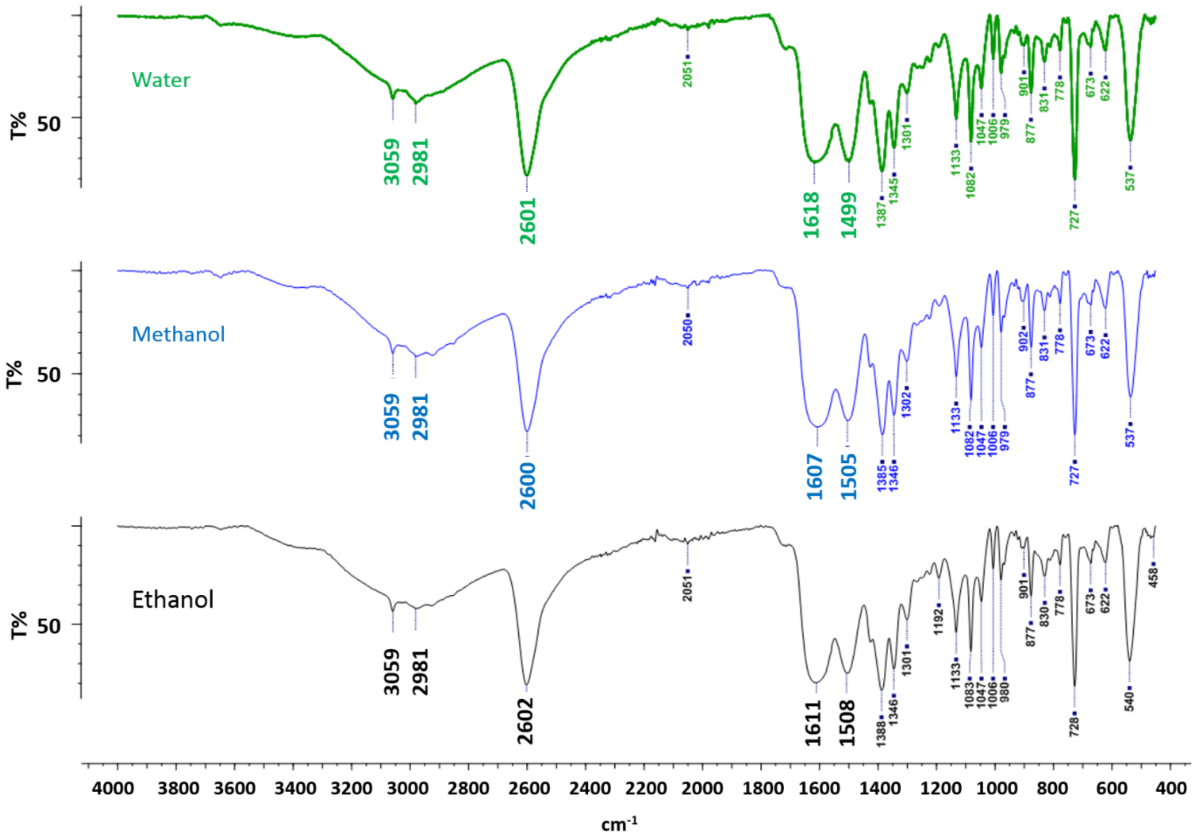

Figure S4. FT-IR spectra of compound **3** derived from evaporation of water (green), methanol (blue) and ethanol solutions (black).

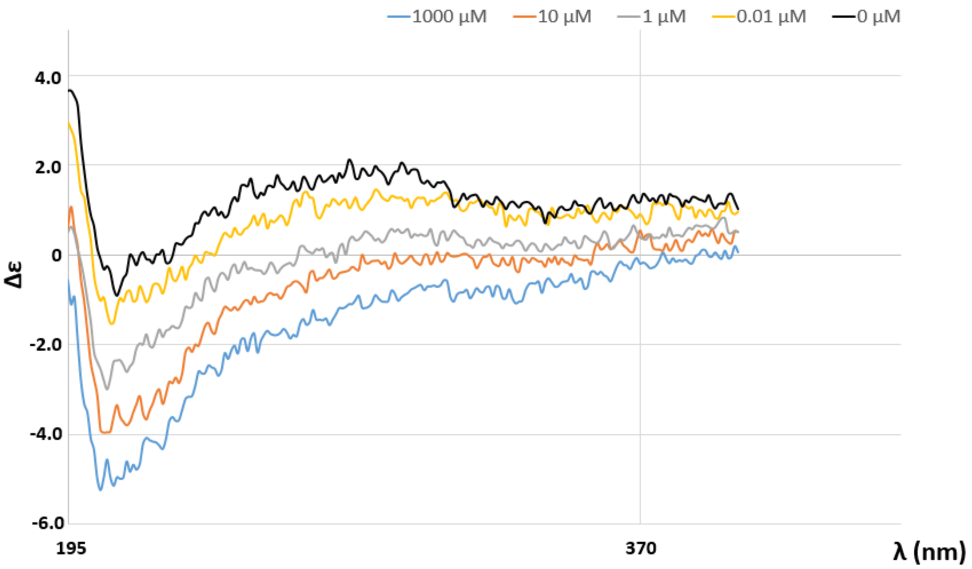

Figure S5. CD spectra of ethanol solutions of 2-acetamido-3-(1,7-dicarba-*closo*-dodecacarboranyl-1-thio)propanoic acid (**3**) showing increasing negative ellipticity with increasing concentrations (at 209 nm).

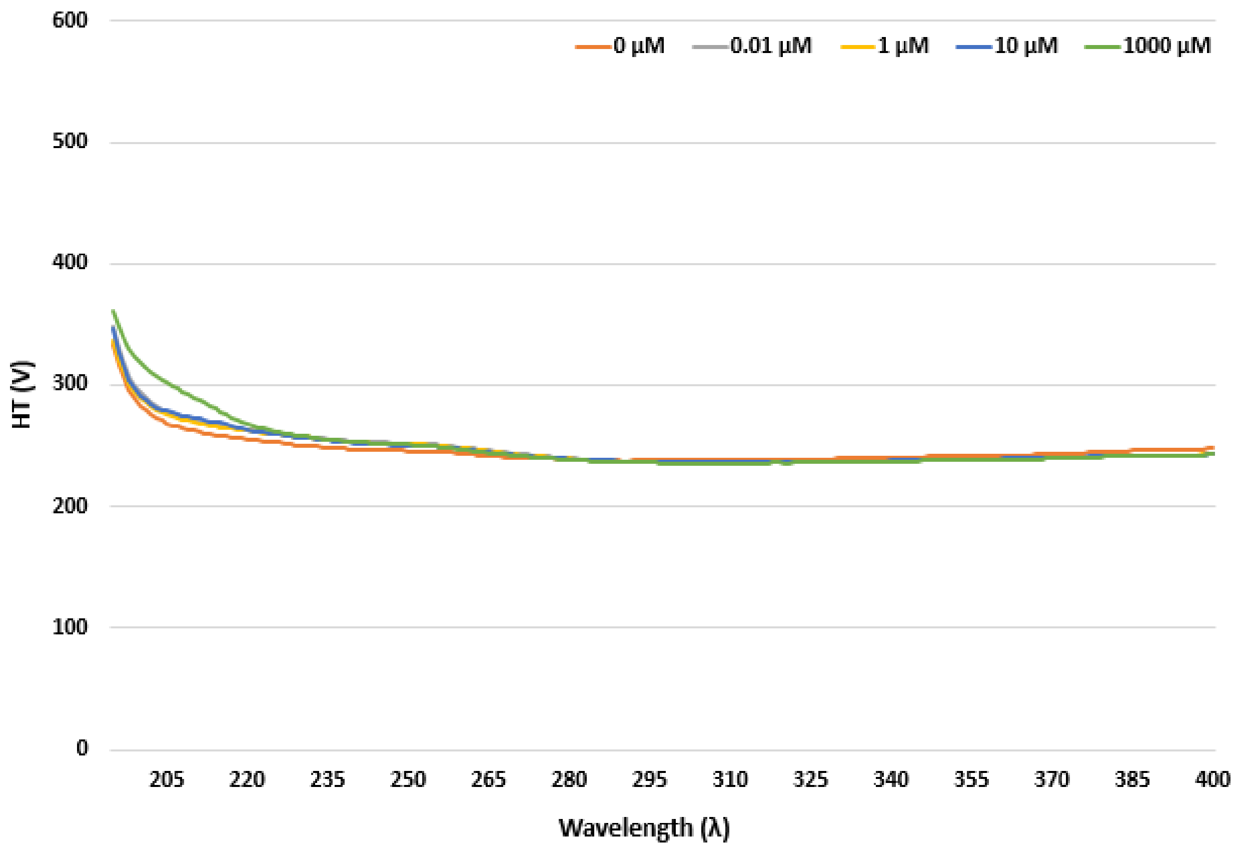

Figure S6. HT curves corresponding to the CD spectra shown in Figure S5.

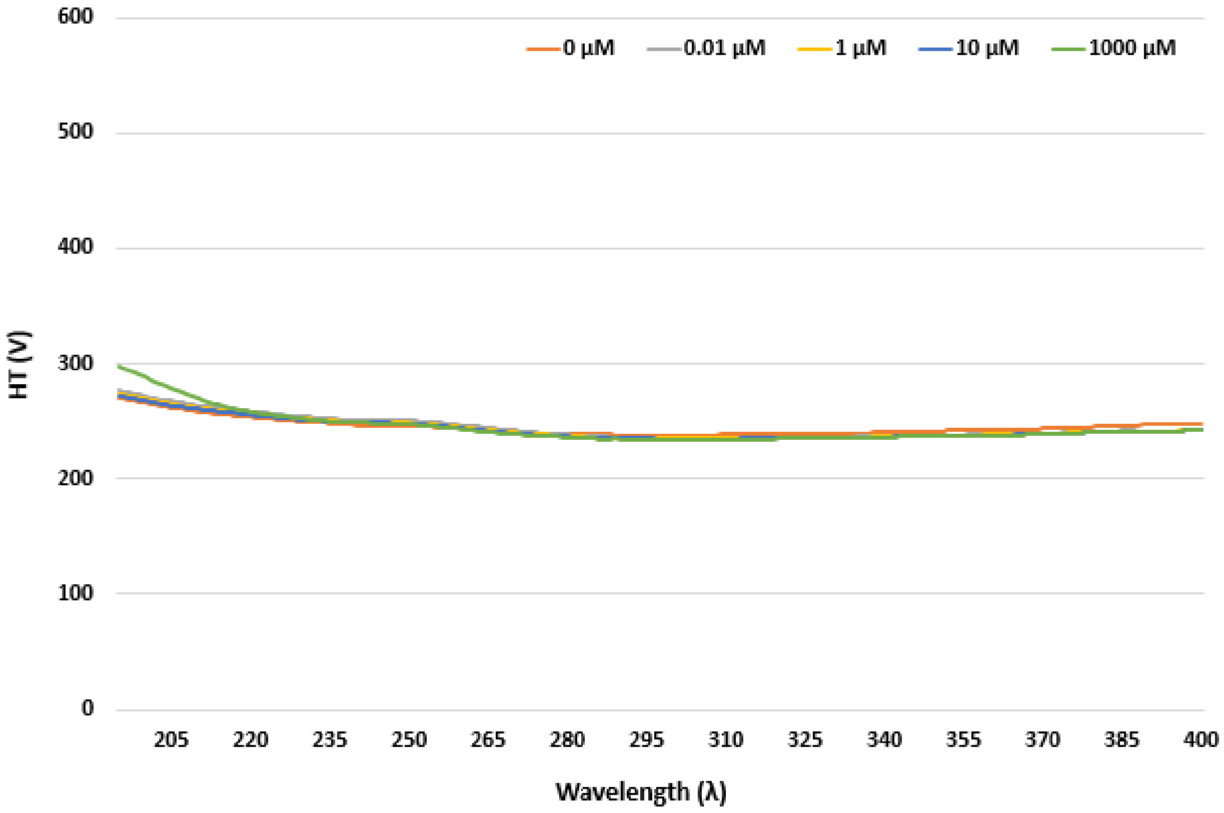

Figure S7. HT curves corresponding to the CD spectra shown in Figure 7.

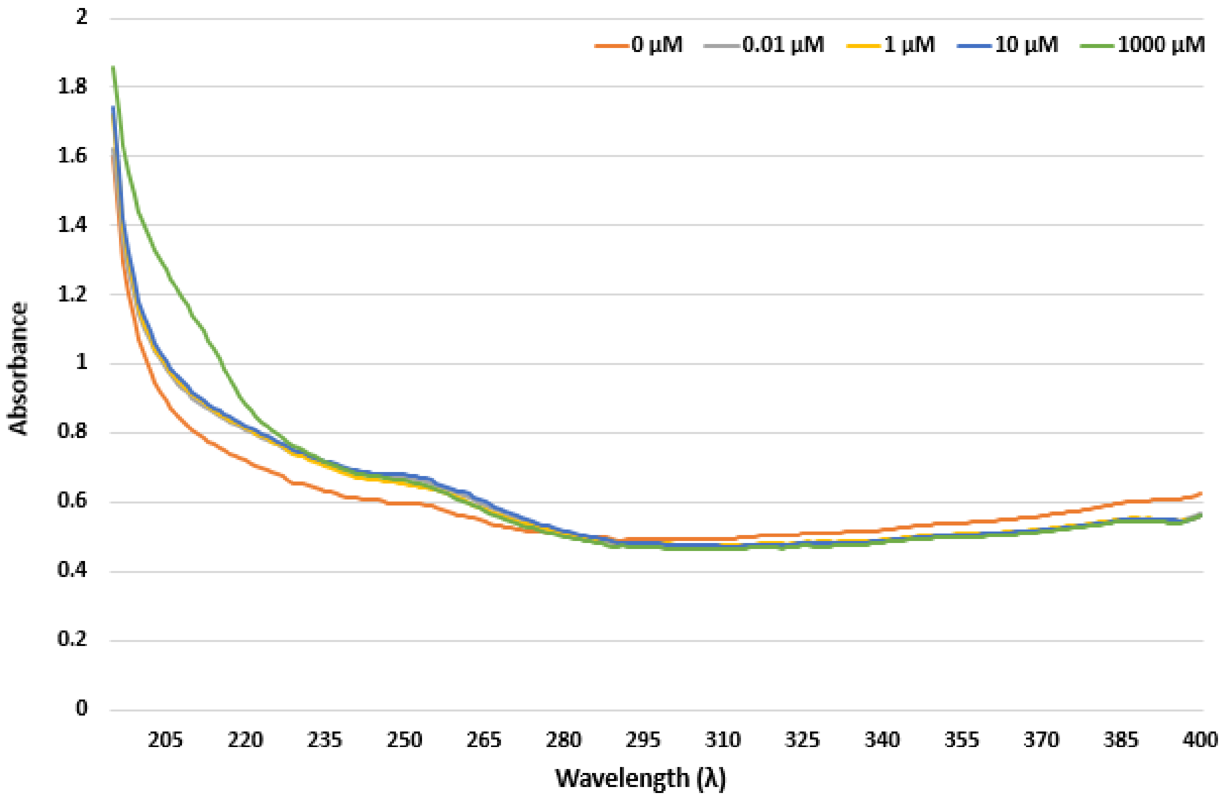

Figure S8. UV absorption spectra of samples corresponding to those whose CD spectra are illustrated in Figure S5 (ethanol solutions of **3**), showing increasing absorbance with increasing concentrations of **3** relative to the control.

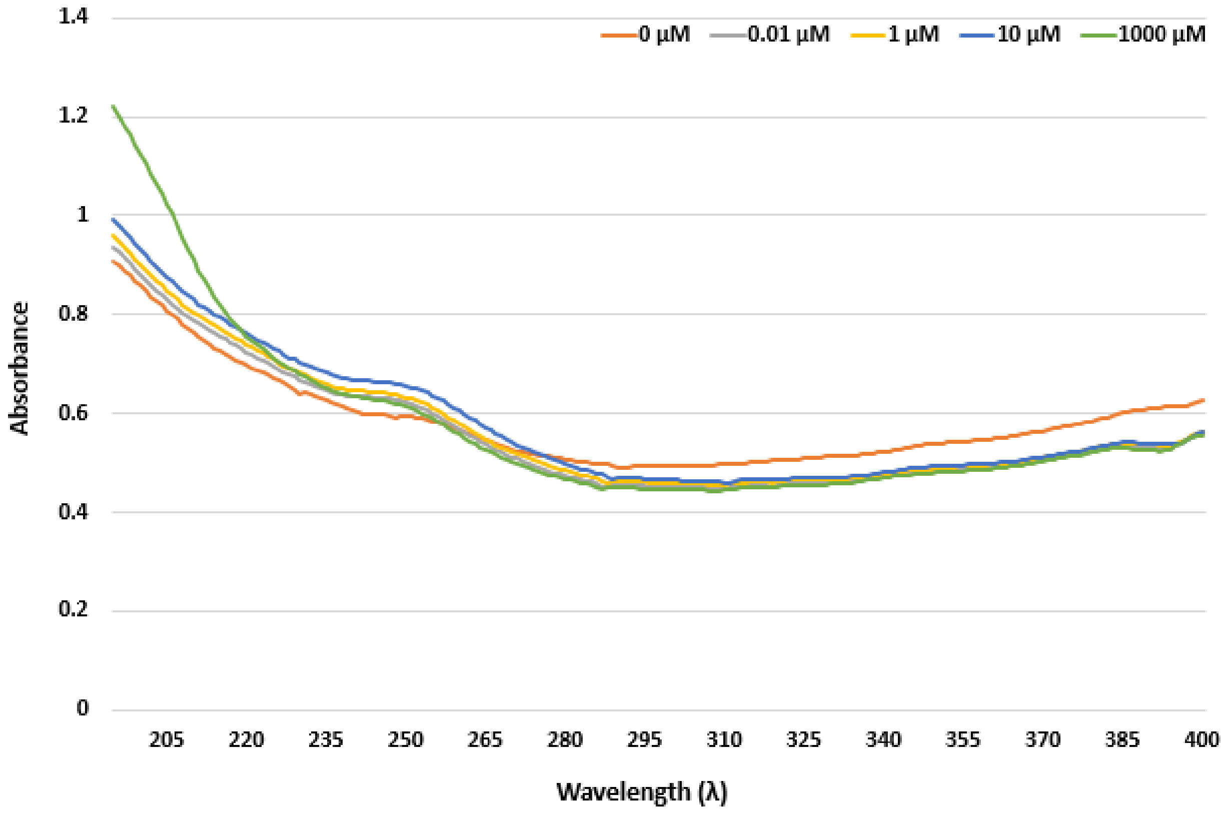

Figure S9. UV absorption spectra of samples corresponding to those whose CD spectra are illustrated in Figure 7 (water solutions of **3**), showing increasing absorbance with increasing concentrations of **3** relative to the control.

General procedure

Data collection was performed on a Bruker D8 VENTURE X-ray diffractometer with PHOTON 100 CMOS detector equipped with a Mo-target X-ray tube (λ = 0.71073 Å) at T = 100(2) K. The single crystal was mounted on a MiTeGen crystal holder (20 mm). A crystal-to-Detector of 60 mm and exposure time of 20 s per frame (0.5° for each frame) were used for data collection. Data reduction and integration were performed with the Bruker software package SAINT (version 8.37A) [1] (SAINT; part of Bruker APEX3 software package (version 2016.9-0): Bruker AXS, 2016). Data were corrected for absorption effects using the empirical methods as implemented in SADABS (version 2016/2) [2] (SADABS; part of Bruker APEX3 software package (version 2016.9-0): Bruker AXS, 2016). The structure was solved by SHELXT [23] and refined by full-matrix least-squares procedures using the Bruker SHELXTL (version 2016/6) [24] software package. All non-hydrogen atoms were refined anisotropically. Hydrogen atoms of N1, O2 and O3 were found in the difference Fourier map and refined independently. All other H-atoms were also included at calculated positions and refined as riders, with $U_{iso}(H) = 1.2\ U_{eq}$ and $U_{iso}(H) = 1.5\ U_{eq}$ for methyl groups.

Crystal growth

The methanol-*d₄* solution of **3** was prepared, filtered, and then transferred into a scintillation vial, and loosely capped. The vial was allowed to stand at 4 °C. Single crystals suitable for X-ray structural measurements were obtained by slow evaporation over a period of 5 weeks at 4 °C.

X-Ray Crystal data of $C_6H_{21}B_{10}NO_3S$ [$(C_5H_{17}B_{10}NO_2S)$ •(CH_4O)]

Table S1. Experimental details

Crystal data	
Chemical formula	$C_5H_{17}B_{10}NO_2S{\cdot}CH_4O$
M_r	295.40
Crystal system, space group	Orthorhombic, *Pbca*
Temperature (K)	100
a, *b*, *c* (Å)	9.4222 (8), 9.5846 (8), 34.794 (3)
V (Å^3)	3142.2 (5)

Z	8
Radiation type	Mo *K*α
μ (mm^{-1})	0.20
Crystal size (mm)	0.21 × 0.16 × 0.03
Data collection	
Diffractometer	Bruker D8 Venture PHOTON 100 CMOS diffractometer
Absorption correction	Multi-scan *SADABS2016*/2 (Bruker,2016/2) was used for absorption correction. Krause, L., Herbst-Irmer, R., Sheldrick G.M. & Stalke D., J. Appl. Cryst. 48 (2015) 3-10
T_{min}, T_{max}	0.922, 1
No. of measured, independent and observed [$I > 2\sigma(I)$] reflections	62413, 4800, 3566
R_{int}	0.100
$(\sin \theta/\lambda)_{max}$ (Å^{-1})	0.715
Refinement	
$R[F^2 > 2\sigma(F^2)]$, $wR(F^2)$, S	0.057, 0.126, 1.12
No. of reflections	4800
No. of parameters	232
No. of restraints	0

H-atom treatment	H atoms treated by a mixture of independent and constrained refinement
$\Delta\rho_{max}$, $\Delta\rho_{min}$ (e Å^{-3})	0.77, −0.76

Computer programs: *APEX3* v.2016.9-0 (Bruker AXS Inc., 2016), *SAINT* V.8.37A (Bruker AXS Inc., 2016), SHELXT 2014/5 (Sheldrick, 2014), *SHELXL2016*/6 (Sheldrick, 2016), Xshell v.6.3.1 (Bruker AXS Inc., 2016).

Table S2. Selected bond distances (Å) and angles (deg.) in the structure of $C_6H_{21}B_{10}NO_3S$ [($C_5H_{17}B_{10}NO_2S$) •(CH_4O)]

Distances		Angles		Angles	
C(1)−B(3)	1.7229(27)	B(2)−C(1)−B(3)	61.34(12)	B(2)−C(2)−B(1)	62.63(12)
C(1)−B(2)	1.7743(31)	B(1)−C(1)−B(3)	111.86(15)	B(6)−C(2)−B(1)	113.88(14)
C(1)−B(1)	1.7804(30)	B(1)−C(1)−B(2)	62.45(12)	B(6)−C(2)−B(2)	62.23(12)
C(1)−B(5)	1.7827(30)	B(5)−C(1)−B(3)	111.23(15)	B(10)−C(2)−B(1)	113.57(14)
C(1)−B(4)	1.7851(30)	B(5)−C(1)−B(2)	112.22(15)	B(10)−C(2)−B(2)	113.81(14)
C(1)−H1X	1.0861(22)	B(5)−C(1)−B(1)	61.25(12)	B(10)−C(2)−B(6)	62.54(12)
		B(4)−C(1)−B(3)	61.56(13)	B(9)−C(2)−B(1)	62.15(11)
C(2)−B(1)	1.7127(27)	B(4)−C(1)−B(2)	112.41(15)	B(9)−C(2)−B(2)	113.84(14)
C(2)−B(2)	1.7157(27)	B(4)−C(1)−B(1)	112.11(15)	B(9)−C(2)−B(6)	113.84(14)
C(2)−B(6)	1.7163(28)	B(4)−C(1)−B(5)	61.57(12)	B(9)−C(2)−B(10)	62.23(12)
C(2)−B(10)	1.7229(27)	H1X−C(1)−B(3)	116.84(128)	S(1)−C(2)−B(1)	113.95(12)
C(2)−B(9)	1.7271(27)	H1X−C(1)−B(2)	117.68(119)	S(1)−C(2)−B(2)	118.84(12)
C(2)−S(1)	1.7916(18)	H1X−C(1)−B(1)	121.52(127)	S(1)−C(2)−B(6)	122.97(12)
		H1X−C(1)−B(5)	122.31(124)	S(1)−C(2)−B(10)	120.42(12)
		H1X−C(1)−B(4)	118.35(122)	S(1)−C(2)−B(9)	114.87(12)

Table S3. Hydrogen bonding distances and angles in the structure of $C_6H_{21}B_{10}NO_3S$ [($C_5H_{17}B_{10}NO_2S$) •(CH_4O)]

D-H	D(D-H)	D(H…A)	<DHA	D(D…A)	A
N1-H1A	0.835	1.964	152.24	2.731	$O1^{i}$
N1-H1D	0.894	1.969	150.91	2.785	$O3^{ii}$
O2-H2X	0.840	1.959	163.14	2.774	$N1^{iii}$
O3- H3X	0.840	1.852	171.18	2.685	O2

Symmetry code: (i) x-1/2, -y+1/2, -z+1; (ii) -x+1, -y+1, -z+1; (iii) -x+3/2, y+1/2, z

1. SAINT; part of Bruker APEX3 software package (version 2016.9-0): Bruker AXS, 2016.

2. SADABS; part of Bruker APEX3 software package (version 2016.9-0): Bruker AXS, 2016.

3. SHELXT; Version 2014/5: G. M. Sheldrick Acta Crystallogr. **2015**, *A71*, 3-8.

4. XL refinement program version 2016/6: **2015**, *C71*, 3-8.
